# Biomarkers of renal recovery after acute kidney injury

**DOI:** 10.5935/0103-507X.20170051

**Published:** 2017

**Authors:** Sérgio Mina Gaião, José Artur Osório de Carvalho Paiva

**Affiliations:** 1 Department of Emergency and Intensive Care, Centro Hospitalar São João, Faculdade de Medicina, Universidade do Porto - Porto, Portugal.; 2 Infection and Sepsis Group - Centro Hospitalar São João, Faculdade de Medicina, Universidade do Porto - Porto, Portugal.

**Keywords:** Acute kidney injury, Renal insufficiency, Renal replacement therapy, Critical care, Intensive care, Biomarkers

## Abstract

Novel biomarkers can be suitable for early acute kidney injury diagnosis and the prediction of the need for dialysis. It remains unclear whether such biomarkers may also play a role in the prediction of recovery after established acute kidney injury or in aiding the decision of when to stop renal support therapy. PubMed, Web of Science and Google Scholar were searched for studies that reported on the epidemiology of renal recovery after acute kidney injury, the risk factors of recovery versus non-recovery after acute kidney injury, and potential biomarkers of acute kidney injury recovery. The reference lists of these articles and relevant review articles were also reviewed. Final references were selected for inclusion in the review based on their relevance. New biomarkers exhibited a potential role in the early diagnosis of acute kidney injury recovery. Urine HGF, IGFBP-7, TIMP-2 and NGAL may improve our ability to predict the odds and timing of recovery and eventually renal support withdrawal. Acute kidney injury recovery requires more study, and its definition needs to be standardized to allow for better and more powerful research on biomarkers because some of them show potential for the prediction of acute kidney injury recovery.

## Abstract (pt)

Novos biomarcadores podem ser apropriados para o diagnóstico precoce da lesão renal aguda e predição da necessidade de diálise. Não é claro se tais biomarcadores podem também desempenhar um papel na predição da recuperação após se ter estabelecido o diagnóstico de lesão renal aguda, ou se podem ajudar na tomada de decisão a respeito do momento de interromper a terapia de suporte renal. Realizamos uma busca nas plataformas *PubMed, Web of Science* e *Google Scholar* por estudos que relatassem a epidemiologia da recuperação renal após lesão renal aguda, sobre fatores de risco para recuperação em comparação a não recuperação após lesão renal aguda, e potenciais biomarcadores de recuperação da lesão renal aguda. A lista de referências destes artigos e os artigos de revisão relevantes foram revisados. As referências finais foram selecionadas para inclusão nesta revisão, com base em sua relevância. Novos biomarcadores revelaram ter um potencial papel no diagnóstico precoce de recuperação da lesão renal aguda. Os níveis urinários do fator de crescimento de hepatócitos, do fator de crescimento semelhante à insulina 7, do inibidor de metalopeptidase 7 e da lipocalina associada com gelatinase de neutrófilos podem aprimorar nossa capacidade de predizer as tendências e a ocasião da recuperação, e eventual remoção do suporte renal. A recuperação da lesão renal aguda demanda mais estudo, e sua definição precisa ser padronizada para permitir melhor e mais potente pesquisa de biomarcadores, pois alguns deles revelam potencial para predição da recuperação de lesão renal aguda.

## INTRODUCTION

The incidence of acute renal failure is still increasing among hospitalized patients^(1-3)^ and ranges from 3 to 25% depending on the criteria used for its definition.^(4)^ Despite significant improvements in our understanding of the pathophysiology of acute kidney injury (AKI)^(5-8)^ and management with multiple clinical treatments, including volume resuscitation, vasoconstrictor and vasodilator therapies,^(9)^ and renal replacement therapy (RRT),^(10)^ the incidence, severity and outcome of AKI have remained similar over recent years.

Some clinical tools are available to quantify AKI, including plasma creatinine, blood urea nitrogen, the presence/absence of urinary casts, the fractional excretion of sodium (FeNa), and urine concentration. However, these markers are of limited use for the early detection of AKI,^(11,12)^ which delays timely interventions. After the introduction of consensus criteria, namely, the Risk Injury Failure Loss End-Stage Renal Disease (RIFLE),^(13)^ Acute Kidney Injury Network (AKIN),^(14,15)^ and, recently, the Kidney Improving Global Outcomes (KDIGO),^(16)^ there has been a positive move toward the use of more standardized definitions in the literature. Unfortunately, and once more, these classifications exclusively base renal dysfunction staging on two inaccurate variables, i.e., plasma creatinine, which is a delayed and unreliable indicator of AKI for many known reasons,^(17-20)^ and urine output, which is influenced by myriad factors including volume status and the use of diuretics. Recently, several plasma and urinary biomarkers have been investigated for the early detection of AKI^(21)^ and have proven to exhibit significant sensitivity and specificity for the purpose of AKI diagnosis and may also play roles in the prediction of short- or long-term outcomes, including death and need for dialysis.^(22)^ However, it remains unclear whether such biomarkers are also suitable for the prediction of recovery after established AKI.

For this review, we searched PubMed, the Web of Science and Google Scholar for reviews and retrospective and prospective studies using keywords or combination of words such as "epidemiology", "renal recovery", "acute kidney injury", "risk factors" and "biomarkers". We included all human studies, and the relevant references lists of these articles were also reviewed. The final references were selected for inclusion in the review on the basis of their relevance.

### Renal recovery after acute kidney injury

The etiology of AKI is commonly referred to as pre-renal (normal tubular and glomerular function but compromised renal perfusion), renal (disease that affect the kidney itself, predominantly the renal tubules, and ischemic renal injury is the most common cause) and post-renal (obstruction of urine release). Unfortunately, even with a correctly oriented treatment for any of these three types of AKI, such as the restoration of normal renal perfusion with intravenous fluids and hemodynamic support for pre-renal AKI, optimization of nutritional and renal support, avoidance of nephrotoxic agents and pharmacological interventions to treat renal AKI,^(23,24)^ and relief of the obstruction for post-renal AKI to prevent irreversible kidney damage, incomplete recovery is a common outcome in patients who survive AKI.^(25)^ However, this anatomic approach has several limitations. Apart from post-renal AKI (i.e., obstructive AKI), the terms do not actually mean very much because pre-renal or renal AKI frequently have similar causes and treatments. In an age in which the term AKI has replaced the term acute renal failure, the concepts of "transient" and "persistent" AKI are probably more useful than pre- or intra-renal AKI.

The cellular response to injury is heterogeneous; some cells undergo necrosis or apoptosis, while others are sublethally injured but are yet able to maintain viability and contribute to a coordinated repair process that restores kidney structure and function.^(26)^ Recently, studies have demonstrated that renal tubular cells enter a period of G1 cell cycle arrest after ischemia^(27)^ or sepsis,^(28)^ which prevents the division of cells with damaged DNA until the DNA is repaired. Despite this heterogeneous response, renal tubular cells have a notable adaptive response to injury. These cells are able to maintain viability, and this feature has been related to some identified genes^(29,30)^ that have been described due to recent advances in functional genomics and cDNA microarray-based technologies. The activation of such pathways represents potential therapeutic targets to lessen AKI severity or to accelerate kidney recovery. However, doubts exist regarding the significance of the metabolic shutdown to "hibernation": Is it a mere failure of kidney function or a protective response to aggression? The fact is that if metabolic activity continues in excess of energy provision, adenosine triphosphate falls, and the cell death pathways are stimulated.^(31)^

A standard definition of AKI renal recovery is essential for providing an accurate account of post-AKI epidemiology; however, this definition does not yet exist. The Acute Dialysis Quality Initiative consensus defines complete renal recovery as a return to baseline creatinine and a partial recovery as an improvement in the RIFLE status of a patient free of dialysis.^(32)^ The fact that only few studies have defined renal recovery according to this recommendation has contributed to a wide range of prevalences of renal recovery across the literature.^(33-38)^ Partial, compared to full, renal recovery is associated with worse long-term survival and a higher incidence of chronic kidney disease (CKD).^(39)^

For prognostication, for intervention planning and for decisions regarding the timing of RRT discontinuation, the ability to distinguish patients who will probably fail to recover kidney function from those who will likely spontaneously recover is of paramount importance.

### Classic markers of acute kidney injury recovery

#### Age

Elderly patients are more prone to develop AKI and to have less successful outcomes.^(40-43)^ It needs to be stressed that most of our understanding of the reparative responses of the kidney result from studies on young healthy animals, and such mechanisms are usually not as efficient in older animals.^(38)^ In a long-term follow-up of 84 patients after AKI, Macedo and colleagues identified age as an independent factor that was associated with non-recovery of renal function.^(44)^ Identically, Srisawat et al. described relations between older age and non-renal recovery in 181 patients with AKI and community-acquired pneumonia^(45)^ and in 76 critically ill patients under RRT.^(46)^ However, in a study of 226 critically ill patients who had AKI requiring RRT, Schiffl did not find any association between age and renal recovery.^(47)^ Similarly, in Moon´s study of 66 patients who developed AKI, there were no differences between the recovery and non-recovery groups with respect to age,^(48)^ and in Alsultan's study of 86 patients who survived to reach the hospital after AKI having required dialysis, the mean age of the patients who were off dialysis was not different from that of the patients who were on dialysis at hospital discharge.^(49)^

#### Chronic kidney disease

Impaired recovery from renal damage may also be a consequence of pre-existing renal disease. Although only two studies suggest that individuals with established CKD recover less successfully from AKI,^(50,51)^ the rationale for the association is solid and well established.^(41,52)^

#### Urine output

The Beginning and Ending Supportive Care for the Kidney study investigators reported that the urine output in the 24 hours prior to the cessation of RRT has good discriminative power to predict the necessity of further RRT.^(53)^ Similarly, Wu et al. found that urine output was one of the most important predictors of early redialysis in patients after weaning from post-operative acute RRT.^(54)^ Recently, in 222 patients who were treated with continuous RRT, urine output during the first 8 hours after the cessation of continuous RRT exerted an independent influence on the outcome of kidney function.^(55)^ However, conflicting data came from a post-analysis of the Veterans Affairs/National Institutes of Health Acute Renal Failure Trial Network, which revealed that urine output, irrespective of diuretic use and as collected on days 1, 7 and 14 from RRT initiation, did not improve the ability to predict renal recovery.^(46)^

#### Fluid accumulation

Patients with higher cumulative fluid balances during RRT exhibited lower renal function recovery in a sub-analysis of the Randomized Evaluation of Normal versus Augmented Level Replacement Therapy (RENAL) trial, which compared doses of RRT.^(55)^ Conversely, Silversides et al.^(56)^ did not find a relationship between fluid balance during RRT and renal function recovery. However, this study only evaluated fluid balance over the first seven days after the initiation RRT and not throughout the entire period of renal replacement therapy. Recently, our group was the first to address this topic as a primary outcome, and in our population, excess fluid accumulation during RRT was associated with non-recovery of renal function among patients with AKI who required RRT.^(57)^

#### Severity score

The severity of illness may help to predict renal recovery from AKI;^(25,57-62)^ however, different studies have used different severity scores. Furthermore, many authors have reported an absence of an association between illness severity and renal recovery.^(48,49,63,64)^

### Type and timing of renal replacement therapy

Recently, in one large retrospective cohort study^(65)^ of critically ill patients with AKI who required renal support, Wald et al. found that the initiation of renal support with continuous RRT (CRRT) is associated with a lower likelihood of chronic dialysis compared with intermittent renal support. Consistent with these findings, Sun et al.^(62)^ documented that a continuous veno-venous hemofiltration was an independent predictor of renal recovery compared to extended daily hemofiltration in septic AKI. Furthermore, a meta-analysis of 50 studies of dialysis dependence after RRT for AKI revealed that an initial renal support with a continuous technique was associated with a lower probability of dialysis dependence than intermittent renal support among AKI survivors.^(66)^

As hypotension^(48)^ and volume overload^(67)^ are documented as factors that are associated with non-recovery of renal function after AKI, CRRT therapy may be the modality of choice for critically ill patients with AKI because it provides better hemodynamic stability and therefore avoids further ischemic insults. However, several studies have reported different results,^(68-70)^ which suggests that we still do not know whether CRRT is better than intermittent renal support in terms of renal recovery. Furthermore, one systematic review and meta-analysis from Karvellas et al. suggested that an early initiation of RRT may have a beneficial influence on dialysis independence.^(71)^ Preliminary data suggest that citrate anticoagulation may be associated with improved AKI recovery that is ultimately due to a reduction in membrane-induced oxidative stress and leucocyte activation.^(72)^

### Acute kidney injury etiology

Some authors maintain that the primary etiology of AKI, defined as pre-renal, renal or post-renal, may influence the chances of renal recovery,^(73,74)^ but inconsistent results have been published.^(44,48)^

### Acute kidney injury severity (RIFLE and plasma/urea)

Identically, the association of AKI severity (i.e., a higher RIFLE score or higher plasma creatinine level) with poor renal recovery has not been uniformly observed across various studies.^(44,45,48,73,75-77)^

Moreover, various clinical variables, such as previous arterial hypertension, cardiovascular instability,^(78)^ low median artery pressure,^(58)^ low diastolic pressure,^(48)^ low central venous pressure,^(58)^ fluid overload,^(79)^ diabetes, higher FeNa, hypomagnesemia, peak creatinine,^(75)^ or even the length of the intensive care unit (ICU) stay and the number of RRT cycles,^(58)^ have been proposed as predictors of renal non-recovery. However, either these associations were described in single unpowered studies, or they were not uniformly observed across studies.

Although some clinical tools and scores exist to predict and stratify AKI, none seem solid and consistent, and therefore, markers to predict recovery from AKI are still lacking. Recently, some studies have assessed urine and plasma biomarkers for this purpose.

### Biomarkers of acute kidney injury recovery

Some major studies are summarized in table 1. Recently, Luk et al.^(77)^ recruited 39 adult patients with pre-existing CKD who presented to the hospital with acute-on-chronic renal failure in stages I or F of the RIFLE classification (excluding patients with anuria) and collected, within the first 24 hours of admission, urine samples to examine neutrophil gelatinase-associated lipocalin (NGAL) and the mRNA expressions of kidney injury molecule-1, interleukin-18 (IL-18), alpha-1-microglobulin (α-1-M), sodium/hydrogen exchanger-3, beta-2 microglobulin and N-acetyl-β-D-glucosaminidase. During the 6-month follow-up, 24 patients experienced complete recovery (defined by authors as the plasma creatinine concentrations falling below 110% of the baseline), 7 experienced partial recovery (defined as plasma creatinine remaining above 110% of the baseline and below 90% of the plasma creatinine at presentation), and 8 experienced no recovery (defined as plasma creatinine remaining above 90% of the plasma creatinine at presentation or whenever the patient became dialysis dependent). The only marker that had a poor but statistically significant correlation with the degree of improvement in renal failure was α-1-M expression (r = 0.387, p = 0.026).

**Table 1:** Summary of human studies on biomarkers of renal recovery after acute kidney injury

Study	Number and type of patients	Biomarker	Timing of biomarkers evaluation	Timing and definition of AKI recovery	Results	
					Conclusions	Statistics
Srisawat et al.^(45)^	181 patients with community-acquired pneumonia and AKI RIFLE-F	Plasma: NGAL and IL-6	First day of RIFLE F classification	Alive and neither requiring renal replacement therapy during hospitalization nor having persistent RIFLE F classification at hospital discharge.	pNGAL predicted failure to recover renal function.	AUC of 0.71 (95% CI 0.61 - 0.81)
					A pNGAL level of 257ng/mL predicted failure to recover	Sensitivity: 68%; specificity: 75%, with positive predictive value of 73% and negative predictive value of 70%.
Srisawat et al.^(46)^	76 critically ill patients who developed AKI and required renal replacement therapy	Urine: NGAL, HGF, cystatin C, IL-18, NGAL/matrix metalloproteinase protein-9 and Creatinine	Days 1, 7, and 14 from RRT initiation	Survival and dialysis independence at 60 days.	uHGF on day 14 predicts AKI recovery	AUC 0.74 (95%CI 0.53 - 0.94)
					The fall of uHGF over the 14 days predicts AKI recovery	AUC 0.74 (95%CI 0.60 - 0.89)
					uNGAL on day 14 predicts AKI recovery	AUC 0.66 (95%CI 0.44 - 0.88)
					The fall of uNGAL over the 14 days predicts AKI recovery	AUC 0.7 (95%CI 0.55 - 0.84)
Moon et al.^(48)^	66 AKI patients with AKI	Urine: NGAL and cystatin C	Every 2 days during 8 days after AKI diagnosis	50% or greater decrease in plasma creatinine from the peak level.	uNGAL at day 0 was a useful predictor of renal recovery	AUC = 0.78, 95%CI 0.65 - 0.9, p < 0.01
					uNGAL level of 348.2ng/mL predicts AKI recovery.	Sensitivity: 0.84, specificity: 0.687, AUC for predicting AKI recovery using uNGAL on days 2, 4, 6 and 8 were 0.813, 0.854, 0.884, and 0.969, respectively
					uNGAL was an earlier marker of recovery compared to plasma creatinine.	
Luk et al.^(77)^	39 patients with pre-existing CKD and AKI RIFLE-I or F	Urine: NGAL, mRNA expression of kidney injury molecule-1, IL-18, α-1-M, sodium/hydrogen exchanger-3, beta-2 microglobulin and N-acetyl-β-D-glucosaminidase	First 24 hours of hospital admission	Evaluation at 6 months. Complete recovery: creatinine falling below 110% of the baseline. Partial recovery: creatinine remaining above 110% of the baseline and below 90% of the creatinine at presentation. No recovery: creatinine remaining above 90% of the creatinine at presentation, or whenever the patient became dialysis dependent.	Urine α-1-M expression had a modest but statistically significant correlation with the degree of improvement in renal failure.	r = 0.387, p = 0.026
Aregger et al.^(82)^	12 critically ill patients	Urine: α-1-M, α-1 antitrypsin, apolipoprotein D, calreticulin, cathepsin D, CD59, IGFBP-7 and NGAL	First day of AKI	Early AKI recovery: less than 7 days; late AKI recovery: more than 7 days.	uIGFBP-7 and uNGAL best predicted renal recovery; uIGFBP-7 was a more accurate predictor of renal outcome than uNGAL.	uIGFBP-7 AUC: 0.74; uNGAL AUC: 0.70).
Meersch et al.^(84)^	26 patients with AKI after cardiac surgery with cardiopulmonary bypass	Urine: TIMP-2*IGFBP7	Preoperatively, 4 hours, 12 hours and 24 hours after coming off cardiopulmonary bypass	Plasma creatinine value at hospital discharge superior or lower than that at baseline.	uTIMP-2*IGFBP7 decline between 4 and 24 hours after surgery served as an accurate marker of renal recovery.	AUC of 0.79 (95%CI: 0.65 - 0.92)
					uNGAL decline between 4 and 24 hours after surgery did not served as an accurate marker of renal recovery.	AUC of 0.48 (95%CI: 0.31 - 0.64).

AKI - acute kidney injury; RIFLE - Risk Injury Failure Loss End-Stage Renal Disease; NGAL - neutrophil gelatinase-associated lipocalin; IL- interleukin; pNGAL- plasma NGAL; AUC - area under the curve; 95%CI - 95% confidence interval; HGF - hepatocyte growth factor; uHGF - urinary hepatocyte growth factor; RRT - renal replacement therapy; uNGAL - urinary NGAL; CKD - chronic kidney disease; α-1-M - alpha-1-microglobulin; IGFBP-7 - insulin-like growth factor-binding protein 7; TIMP-2 - metallopeptidase inhibitor.

In a *post hoc* analysis of a multicenter prospective study of community-acquired pneumonia,^(80)^ Srisawat et al. assessed plasma NGAL (pNGAL) and interleukin-6 (IL-6) as markers for the prediction of recovery from AKI.^(45)^ Acute kidney injury recovery was defined as the patient being alive and neither requiring RRT during hospitalization nor having a persistent RIFLE F classification at hospital discharge. Samples were collected on the first day of RIFLE-F classification. Of the 181 patients, 93 (51.4%) experienced renal recovery. The median pNGAL concentration was significantly lower in the recovery group (165ng/mL (IQR 113 - 266)) than in the non-recovery group (371ng/mL (IQR 201 - 519)) (p < 0.001). There was no difference in plasma IL-6 between the recovery and non-recovery groups. There was also a poor correlation between pNGAL and IL-6 (correlation coefficient = 0.31). Among the survivors (n = 138), pNGAL predicted the failure to recover renal function with an area under the curve (AUC) of 0.71 (95%CI 0.61 - 0.81), and a pNGAL level of 257ng/mL predicted the failure to recover with a sensitivity of 68%, a specificity of 75%, and positive and negative predictive values of 73% and 70%, respectively.

Moon et al. measured urinary NGAL (uNGAL) and cystatin C levels every 2 days for 8 days in 66 AKI patients.^(48)^ AKI was defined as a 50% or greater increase in plasma creatinine from baseline. AKI recovery was defined as a 50% or greater decrease in plasma creatinine from the peak level. The primary endpoint of the study was recovery from AKI. At day 0, there were no differences in plasma creatinine, BUN or urine cystatin C between the AKI patients in the recovery (n = 33) and the non-recovery (n = 33) groups. However, there was a significant difference in uNGAL between the two groups from day 0 (297.2 *versus* 407.6ng/mL, p = 0.025) and throughout the study period until day 8 (123.7 *versus* 434.3, p < 0.001). Urine NGAL at day 0 was a useful predictor of renal recovery (AUC = 0.78, 95%CI 0.65 - 0.9, p < 0.01), and for a cut-off value of 348.2ng/mL, the sensitivity and specificity were 0.84 and 0.687, respectively. The AUCs for predicting AKI recovery using uNGAL on days 2, 4, 6 and 8 were 0.813, 0.854, 0.884, and 0.969, respectively. Urine NGAL was a better earlier marker of recovery from AKI compared with plasma creatinine.

The Biological Markers of Recovery for the Kidney (BioMarK) study^(46)^ was conducted as an ancillary to the ATN study.^(81)^ Urine samples were collected on days 1, 7, and 14 from 76 patients who developed AKI and required RRT to explore whether uNGAL, urinary hepatocyte growth factor (uHGF), urinary cystatin C (uCystatin C), urinary IL-18 (uIL-18), uNGAL/matrix metalloproteinase protein-9 and urinary creatinine could predict renal recovery. Recovery of renal function was defined by survival and dialysis independence at 60 days. Exactly half of the patients (n = 38) recovered renal function, and 26 of these patients experienced complete recovery. The patients who recovered had higher uCystatin C levels on day 1 and lower uHGF levels on days 7 and 14. For predicting recovery, the AUC for uHGF on day 14 and for the fall of uHGF over the first 14 days were 0.74 (95%CI 0.53 - 0.94) and 0.74 (95%CI 0.60 - 0.89), respectively. The AUC for uNGAL on day 14 and for the fall of uNGAL over the first 14 days were 0.66 (95%CI 0.44 - 0.88) and 0.7 (95%CI 0.55 - 0.84), respectively.

A recent proteomic approach identified urinary biomarkers that could predict AKI recovery by comparing 12 critically ill patients with early AKI recovery (less than 7 days) with 12 patients with late recovery (more than 7 days) or no recovery.^(82)^ From the 8 candidate urinary biomarkers in the study (i.e., α-1 microglobulin, α-1 antitrypsin, apolipoprotein D, calreticulin, cathepsin D, CD59, insulin-like growth factor-binding protein 7 (IGFBP-7), and NGAL), only urinary IGFBP-7 (uIGFBP-7) and uNGAL adequately predicted renal recovery (uIGFBP-7 AUC: 0.74; uNGAL: 0.70) and were associated with the duration of AKI. IGFBP-7 was a more accurate predictor of renal outcome than uNGAL. These results support those of the study by Sapphire.^(83)^ In this large (n = 728) multicenter study of cell cycle arrest, uIGFBP-7 and metallopeptidase inhibitor 2 (TIMP-2) were both markers of moderate-to-severe AKI (KDIGO stage 2 - 3) within 12 hours of sample collection, and additionally, high values of multiplication of the 2 markers (urinary IGFBP*TIMP-2) doubled the risk of major adverse kidney events, which were defined by the authors as death, the use of RRT or the persistence of renal dysfunction; these findings suggest a role for this combination of biomarkers as predictors of recovery.

Furthermore, in 50 patients who were at risk for AKI and were undergoing cardiac surgery with cardiopulmonary bypass, Meersch et al.^(84)^ investigated whether urinary TIMP-2*IGFBP7 could predict renal recovery from AKI, which was defined as a plasma creatinine value at hospital discharge equal or lower than that at baseline. Urine samples were collected preoperatively on the day of surgery and at 4 hours, 12 hours and 24 hours after coming off the cardiopulmonary bypass. Of the 50 patients, 26 developed AKI as defined by the KDIGO criteria. TIMP-2*IGFBP7 served as a sensitive and specific marker of AKI early after cardiac surgery, and the decline between 4 and 24 hours after surgery served as an accurate marker of renal recovery with an AUC of 0.79 (95%CI: 0.65 - 0.92). The AUC for the difference between the two uNGAL values was 0.48 (95%CI: 0.31 - 0.64).

## COMMENTS

AKI research has progressed substantially since consensus definitions for AKI and its severity were formulated. However, no AKI recovery definition was created, and the significant differences between studies regarding this concept make research on epidemiology, prognostication and ultimately interventions in the recovery phase of AKI extremely difficult. The identification of patients who are at high risk for failure to recover has important implications for their immediate and long-term care, namely, the avoidance/minimization of nephrotoxins or early referral to a nephrologist. Furthermore, some recent advances in recovery enhancers, such as formoterol,^(85)^ atrasentan^(86)^ or mesenchymal stem cells^(87-89)^ stress the need to identify *who* (probably patients with the potential for recovery) may benefit from such treatments and when (probably as soon as renal recovery is expected) these patients may be expected to do so.

Although age, CKD, systemic severity scores, AKI severity, urine output and early, continuous renal support using citrate anticoagulation have been associated with a greater chance of renal function recovery, the results are not consistent across different studies, and the influence of their use in clinical practice is almost irrelevant.

New biomarkers have exhibited possible roles in the early and accurate diagnosis of AKI recovery. Indeed, urine HGF, IGFBP-7, TIMP-2 and NGAL may improve our ability to predict the odds and timing of recovery and therefore predict renal support withdrawal. However, studies and cases are few, and additional and more powerful research is needed. Furthermore, a more comprehensive physiological understanding of the AKI-to-CKD transition will be useful in the search for which marker can be more specific in terms of recovery and can be tested for that purpose.
